# Evaluation of the otolith function using c/oVEMPs in patients with Ménière’s disease

**DOI:** 10.1186/s40463-016-0152-4

**Published:** 2016-06-21

**Authors:** Liang Chen, Hui Xu, Wu-qing Wang, Qing-quan Zhang, Qiao-ying Lv, Xi-cheng Song

**Affiliations:** Otorhinolaryngology Head and Neck Surgery Department, Affiliated Yantai Yuhuangding Hospital of Qingdao University Medical College, Yantai City, Shandong Province China; Stomatology Department, Affiliated Yantai Yuhuangding Hospital of Qingdao University Medical College, Yantai City, Shandong Province China; Otorhinolaryngology Head and Neck Surgery Department, Affiliated Eye & ENT Hospital of Fudan University, Shanghai, China; Otorhinolaryngology Department, Affiliated Yantai Yuhuangding Hospital of Qingdao University Medical College, Yantai City, Shandong Province China

**Keywords:** Cervical/ocular vestibular evoked myogenic potentials (c/oVEMPs), Ménière’s disease (MD), Otolith

## Abstract

**Background:**

Cervical and ocular vestibular evoked myogenic potentials (c/oVEMPs) reflect otolith function. Up-to-date, there are no published reports on the systemic evaluation of otolith function in Ménière’s Disease (MD) nor are there any reports on the differences in VEMPs between patients with early and late stage MD. The aim of this study was to evaluate the difference in c/oVEMPs between patients with MD and normal controls, as well as between patients with early and late stage MD.

**Methods:**

Thirty patients with unilateral MD and thirty healthy subjects (as normal controls) were prospectively enrolled. c/oVEMPs using 500 Hz tone-burst stimuli were performed. VEMP tests were repeated 3 times on each subject to ensure reliability and reproducibility of responses. VEMPs were defined as present or absent. Abnormal VEMP was defined by lack of VEMP response.

**Results:**

In the control group, abnormal cVEMPs and oVEMPs responses were detected in 6.67 and 3.34 % respectively. In MD patients (20 with early stage MD [ES-MD], 10 with late stage MD [LS-MD]), abnormal cVEMPs and oVEMPs responses were detected in 40 and 16.7 % respectively. More patients with MD showed abnormal responses in c/oVEMPs as compared to the control group (*p* < 0.05). cVEMPs was more often abnormal as compared to oVEMPs in MD patients (*p* < 0.05). There was a significant difference in abnormal cVEMP responses between ES-MD patients (25 %) and LS-MD patients (70 %) (*p* < 0.05). Difference in abnormal oVEMP responses (ES-MD, 5 %; LS-MD, 40 %) was significant (*p* < 0.05).

**Conclusion:**

An increased occurrence of abnormal c/oVEMP recordings appeared in MD patients, possibly as a result of hydrops of the otolith. cVEMPs were more often abnormal in MD patients as compared to oVEMPs, suggesting that saccular dysfunction may be more common than utricular dysfunction. Furthermore, o/cVEMP abnormalities in the LS-MD group were significantly higher than those in the ES-MD group, suggesting the trend that otolith damage is gradually increasing with the aggravation of cochlear injury in MD.

## Background

By stimulating the ear with air-conducted sound (ACS) or bone-conducted vibration (BCV) stimuli, vestibular-evoked myogenic potential (VEMP) can be recorded on the contracted neck muscles, so-called cervical VEMP (cVEMP), and on the extraocular muscles, termed ocular VEMP (oVEMP). In 1992 and 1994, cVEMPs was first described by Colebatch and Halmagyi [1, 2], who measured electromyographic (EMG) activity from the sternocleidomastoid (SCM) muscles following vestibular stimulation with clicks. In 1995, Halmagyi et al. [3] elicited cVEMPs by tapping the forehead. The responses had the same biphasic waveform as the AC cVEMPs and were vestibular-dependent. In 2000, Sheykholeslami et al. [4] recorded cVEMPs using BC sound delivered to the mastoid bone with a clinical bone conductor. In 2005 and 2007, Rosengren [5] and Todd [6] recorded the short latency potentials from around the eyes and demonstrated that it can also be recorded from the extraocular muscles. It was recently reported that ocular VEMPs (oVEMPs) are produced by synchronous activity in the extraocular muscles in response to stimulation, including sound [7]. A more recent study reported that oVEMPs in response to air-conducted sound (ACS) reflect functions of different parts of the vestibular labyrinth from cVEMPs in response to ACS; that is, oVEMPs predominantly reflect utricular functions while cVEMPs reflect saccular functions [8].

Prosper Ménière described MD in 1861, but a diagnostic test with high specificity and sensitivity still has not been developed. Furthermore, even the histological findings are not directly related to the symptoms and clinical course [9]. Clinicians make a diagnosis based on the symptoms and the results of a hearing test. In 1995, the American Academy of Otolaryngology-Head and Neck Surgery (AAO-HNS) proposed diagnostic criteria for MD and a staging system based on hearing function measured by pure tone threshold at 0.5, 1.0, 2.0, and 3.0 kHz [10]. Patients with MD present fluctuating hearing and vertigo. Therefore, it is important to identify irreversible damage among the fluctuating symptoms. Currently, irreversible damage from MD is measured solely on the basis of hearing impairment. However in the conventional staging system, intact vestibular function is usually evident in later stage MD [11]. One possible hypothesis is that the caloric test does not reflect vestibular loss in MD [12]. A vestibular function test that reflects functional loss in MD is clearly required. Because the otolith is anatomically close to the cochlea, there is a possibility that its functional loss may occur just after the cochlear functional loss. Okuno et al. [13] reported on the incidence of endolymphatic hydrops in each department of the labyrinth. Twenty-two temporal bones from deceased patients with MD were examined. The authors reported cochlear hydrops in all temporal bones; 86.5 % had saccular hydrops, 50 % had utricular hydrops and 36.4 % had hydrops in the semicircular canal [14].

Based on this pathological background, VEMP can be used as a test of the otolith organ and peripheral vestibular function [2]. Several studies have investigated the correlation of VEMP with hearing impairment in MD. Young et al. [15] defined the interaural amplitude difference (IAD) ratio in 40 patients, and reported a correlation of the IAD ratio of VEMPs with the conventional stage of MD. De Waele et al. [12] reported that saccular impairment correlates with low-frequency hearing loss. However, other studies did not report a significant difference in VEMPs from MD patients compared to normal subjects [16, 17]. Thus, the clinical role of VEMP in MD remains debatable. Furthermore, the systemic evaluation of otolith damage in MD patients has never been reported. Up to date, there are no reports on the differences in VEMPs between early stage MD (ES-MD) patients (I, II, III stage) and late stage MD (LS-MD) patients (IV stage). The aim of this study is to determine the diagnostic value of VEMP in MD by evaluating the difference in c/oVEMPs between patients with MD and controls, as well as between patients with ES-MD and LS-MD. Through this analysis, we tried to confirm the role of VEMP as a new evaluation test.

## Methods

### Subjects

Ethical approval was received from the Ethics Committee of Affiliated Yantai Yuhuangding Hospital of Qingdao University Medical College. Between January 2013 and January 2015, 30 consecutive patients with definite unilateral Menière’s disease were prospectively enrolled from the Dizziness Clinic of Affiliated Yantai Yuhuangding Hospital of Qingdao University Medical College. Audiometric evaluation was performed at frequencies of 500, 1000, 2000 and 3000 Hz, and patients were grouped according to AAO-HNS guidelines: stage I, 0–25 dB hearing level (HL); stage II, 25–40 dB HL; stage III, 41–70 dB HL, and stage IV worse than 70 dB HL. Patients were divided into two groups according to the stage- 20 early stage patients (ES-MD group, I, II, III stage) and 10 late stage (LS-MD group, IV stage). The diagnosis and periodization of MD patients was according to the criteria for definite MD recommended in 1995 by the AAO-HNS Committee on Hearing and Equilibrium [10].

Patients who underwent endolymphatic sac surgery or who were treated with an intratympanic injection of gentamicin before enrollment were excluded from the analysis. In addition, patients with a history of a brain tumor or vestibular schwannoma, or any neurological, psychiatric, or significant medical disease were excluded. The control subjects were all volunteers from our normal outpatient clinic who had no otological disease. Patients with a history of hearing loss, other vestibular disorders and >60 years old were excluded. Informed consent was obtained from each subject according to the Declaration of Helsinki.

### cVEMPs

Cervical vestibular evoked myogenic potentials (cVEMPs) testing was performed on both sides for all patients and controls. In the cVEMPs test, all subjects were placed in a sitting position and asked to rotate their head away from the stimulated side so as to record electromyographic activity over tonically activated sternocleidomastoid (SCM) muscles. Surface EMG activity was recorded with superficial electrodes placed on the middle third of the SCM, with the reference electrode placed on the upper third of the sternum and the ground electrode on the middle of the forehead. Using a Bio-Logic Navigator Pro, 90 dBnHL 500 Hz tone bursts were presented through headphones, and the EMG signal was amplified and bandpass filtered (30–1500 Hz). The analysis window was 100 ms wide and responses to 120 stimuli were averaged. cVEMP tests were repeated 3 times on each subject to ensure reliability and reproducibility of responses. The amplitude of the first positive–negative peak (P13–N23) was recorded. Absence of a meaningful wave form with p13 and n23 was defined as ‘no response’. Abnormality was strictly defined as a cVEMP pattern of ‘no response’.

### oVEMPs

Ocular vestibular evoked myogenic potentials (oVEMPs) testing was performed on both sides for all patients and controls. In the oVEMPs test, all subjects assumed a sitting position and the subject was instructed to look superomedially at a small fixed target 1 m from the eyes. The visual angle was approximately 30°, which has been found to elicit the largest responses compared with other eye positions [18]. The active electrodes were placed on the face, oriented vertically and approximately 1 cm below the center of the lower eyelid just inferior to the contralateral eye for sound stimulation. The reference electrode was placed about 1 cm below the active electrode on the cheek, and the ground electrode was placed on the forehead. Each subject’s eyes remained fixed on the target throughout the test. Using a Bio-Logic Navigator Pro, 95 dBnHL 500 Hz tone bursts were presented through headphones, and the EMG signal was amplified and bandpass filtered (10–300 Hz). The analysis window was 100 ms wide and responses to 120 stimuli were averaged. oVEMP tests were repeated 3 times on each subject to ensure reliability and reproducibility of responses. The initial negative–positive biphasic waveform comprised peaks N1 and P1. We analyzed the waveforms of N1 and P1 at the maximal intensity of stimulation. Abnormality was strictly defined as an oVEMP pattern of ‘no response’.

### Statistical analysis

A Fishers exact or Chi-squared test was used to analyze the statistical significance of the inter-group difference in the number of non- cVEMPs and oVEMPs responders in the MD and controls groups. A *p* value of < 0.05 indicated statistical significance.

## Results

The ages ranged from 35 to 54 years (mean 45 years, 12 men and 18 women) in patients with MD. The control group consisted of 30 normal subjects (10 men and 20 women; mean age 40 years; age range, 30–60 years). The Pure-Tone-Average (PTA) of ES-MD and LS-MD groups were 45 and 80 dBnHL respectively. Demographic data for MD and control groups are summarized in Table 1. There were no significant differences in age and sex ratio between the two groups (*p* > 0.05). Testing of VEMPs was performed on both sides in all MD patients and controls. No patient with MD showed bilateral abnormalities, so only ipsilesional data are presented. In the control group, abnormal cVEMP responses were detected in 2 of 30 (6.67 %) subjects and abnormal oVEMP responses were detected in 1 of 30 (3.34 %) subjects (Table 2).

**Table 1:** Demographic features of subjects in MD and control groups

Group feature	Control	MD	
		ES	LS
Number	30	20	10
Age*(M ± SD)	40 ± 8.0	42 ± 6.0	47 ± 7.0
Sex(M:F)*	10:20	8:12	4:6
PTA(M ± SD)	Normal	45 ± 6.5	80 ± 4.0

*MD* Ménière’s disease. *p > 0.05. *ES* early stage group in MD, *LS* late stage group in MD, *PTA* pure-tone-average

**Table 2:** The abnormal c/oVEMP responses details in control and MD groups (ES, LS)

Groups (cases)	Abnormal		Normal		Percent	
	cVEMP	oVEMP	cVEMP	oVEMP	cVEMP	oVEMP
Control(30)	2	1	28	29	6.67 %	3.34 %
ES-MD(20)	5	1	15	19	25 %	5 %
LS-MD(10)	7	4	3	6	70 %	40 %
MD(30)	12	5	18	25	40 %	16.7 %

*MD* Ménière’s disease, *ES-MD* early stage group in MD, *LS-MD* late stage group in MD; In cVEMPs, MD *VS* Control: (*p* < 0.05), ES *VS* LS: (*p* < 0.05); In oVEMPs, MD *VS* Control: (*p* < 0.05), ES *VS* LS: (*p* < 0.05); In MD, cVEMP *VS* oVEMP: (*p* < 0.05)

### cVEMP abnormalities in MD patients

Abnormal cVEMP responses were detected in 12 of 30 (40 %) MD subjects. More patients with MD showed abnormal responses in cVEMPs as compared to the controls (*p* < 0.05). In cVEMPs testing, abnormalities were detected in 5 of 20 (25 %) in the ES-MD group and in 7 of 10 (70 %) subjects in the LS-MD group; the difference between the two groups was significant (*p* < 0.05) (Table 2).

### oVEMP abnormalities in MD patients

Abnormal oVEMP responses were detected in 5 of 30 (16.7 %) subjects in MD group. More patients with MD showed abnormal responses in oVEMPs as compared to the controls (*p* < 0.05). In oVEMPs testing, abnormalities were detected in 1 of 20 (5 %) in the ES-MD group and in 4 of 10 (40 %) subjects in the LS-MD group; the difference between the two groups was significant (*p* < 0.05) (Table 2).

### Comparison of cVEMPs with oVEMPs in MD patients

Abnormal cVMEPs responses were detected in 12 of 30 (40 %) subjects in MD group while abnormal oVMEPs responses were detected in 5 of 30 (16.7 %). The abnormal results for cVEMPs showed a higher percentage than those for oVEMPs in MD patients (*p* < 0.05) (Table 2).

## Discussion

The main objective in this study was to report c/oVEMPs findings in MD patients and to verify some clinical characteristics of MD in VEMPs. We evaluated the function of otolith by measuring c/oVEMPs. At present, cVEMPs which are evoked by air-conducted sound (ACS), are widely used to evaluate the function of saccule and inferior vestibular nerve by recording the inhibitory potential from the SCM. Curthoys et al. [7] and Shin et al. [19] reported that the oVEMP evoked by ACS may be predominantly mediated by the superior vestibular nerve due to the activation of the utricular receptors. Ménière’s disease (MD) is an idiopathic syndrome characterized by recurrent vertigo, hearing loss, ear fullness and tinnitus [18]. Its pathological basis is hydrops and dilatation of the endolymphatic spaces involved in both hearing and balance. Endolymphatic hydrops leads to distortion of the membranous labyrinth or rupture of Reissener’s membrane [13, 20]. Hydrops occurs most often in the cochlea, with the otolith as the second most frequent site for endolymphatic hydrops [21]. The hydrops process at the level of the otoliths could therefore be detected using c/o VEMPs in MD patients. With regards to the amplitude and latency values of VEMPs, there are many factors that can affect these values such as basic muscle activity, patient’s position, and general conditions [22, 23]. Because of their non-specific value in VEMPs testing, we used a qualitative approach to VEMPs results. Our definition for abnormal VEMP is an absence of VEMP response.

In our study, we found that patients with MD showed higher rate of abnormal responses in c/oVEMPs by stimulation on their affected side than the controls. The incidence of abnormal response of o/cVEMP in our study was 40 and 16.7 % respectively. The abnormal incidence of cVEMPs is a little higher than the results reported by Egami et al. [24], who studied 114 cases of unilateral MD by cVEMPs and found that 34 (29.8 %) showed abnormal click-VEMPs solely on the affected side (decreased responses in 8 patients; absent responses in 26 patients). In 2015, Salviza et al. [25] researched the frequency-associated VEMP responds in MD diseases, the incidence of no oVEMP responses to 500-Hz ACS were 23 % in affected ears of patients with MD which the abnormal incidence of oVEMPs is similar to our result.

In cVEMPs testing, abnormalities were detected in 25 % of subjects in the ES MD group and in 70 % of subjects in the LS MD group. There was a higher rate of abnormality in the LS MD group, the difference between the two groups was significant. This suggests that LS MD patients may have more saccular damage result in gradual increases with the aggravation of cochlear injury in MD. In oVEMPs testing, abnormalities were detected in 5 % of subjects in the ES MD group and in 40 % of subjects in the LS MD group. Abnormal response rate was significantly higher in LS MD patients than ES MD patients. This suggests that LS MD patients may have more utricular damage which also gradually increases with the aggravation of cochlear injury in MD. In 2013, Kim et al. [26] compared interaural amplitude difference (IAD) ratio of VEMP with the MD staging system to evaluate the clinical usefulness of VEMP in predicting the disease stage. There was a tendency toward a decreasing response of VEMP, but this trend was not statistically significant. We used a qualitative approach defining an absence of VEMP response as abnormal to VEMPs results and verified the trend that otolith damage is gradually increasing with the aggravation of cochlear injury in MD.

In our study, there was a higher rate of abnormal cVEMP results than oVEMP results in MD patients. These findings support the possibility that saccular function in MD patients is more heavily damaged than utricular function. Konishi et al. [27] observed that the utriculo-endolymphatic (UE) valve opens for several days after hydrops formation begins and then closes due to the compression caused by increasing hydrops. The UE valve exists at the junction between the utricle and utricular duct. The endolymph can easily flow in the direction from the utricular duct to the utricle but not in the reverse direction thus concluding the role of the UE valve is to maintain pressure in the utricle and semicircular canals [28]. An acute pressure reduction in the saccule can collapse the pars superior (saccule and cochlear duct) but not the utricle, therefore endolymph pressure in the utricle can be maintained independently of the pressure in the pars superior [29]. Our findings of more damage in saccule than utricule can also support this theory found by Wen et al. [29].

VEMPs is a good method for evaluation of otolith function; however this test requires specialized equipment and complicated procedures for separate analyses of utricular and saccular function as the VOR gain reduction is under the influence of both utricular and saccular dysfunction, in addition to semicircular canal-otolith interaction. In the present study, abnormalities in cVEMPs was frequently detected which is consistent with the suggested underlying pathology of MD, namely hydrops of the saccular prior to the utricular. Those ears showing abnormal cVEMPs as well as oVEMPs might have severe hydrops changes causing dysfunction of the utricle and the saccule.

Considering the controversy in the stability and repeatability of c/oVEMPs, we defined abnormal c/oVEMPs as absent responses in our study. As a result, we did not analyse the latency and amplitude of the waves in c/oVEMPs. The major limitation of our study is the small number of MD patients. Another limitation is the average age of our study subjects were under 60 years and the incidence of MD is higher in the elderly resulting in a potential bias in the research. In future research, a larger sample size will be obtained such that quantitative analysis of otolith function in MD patients can be performed.

## Conclusion

An increased occurrence of abnormal c/oVEMP recordings appeared in MD patients, possibly as a result of hydrop of the otolith. cVEMPs were more often abnormal in MD patients as compared to oVEMPs, suggesting that saccular dysfunction may be more common than utricular dysfunction. Furthermore, o/cVEMP abnormalities in the LS-MD group were significantly higher than those in the ES-MD group, suggesting the trend that otolith damage is gradually increasing with the aggravation of cochlear injury in MD.

## Abbreviations

c/oVEMPs, cervical and ocular vestibular evoked myogenic potentials; ES-MD, early stage MD; LS-MD, late stage MD; MD, Ménière’s disease; PTA, pure-tone-average
